# Mural Cell Associated VEGF Is Required for Organotypic Vessel Formation

**DOI:** 10.1371/journal.pone.0005798

**Published:** 2009-06-04

**Authors:** Lasse Evensen, David R. Micklem, Anna Blois, Sissel Vik Berge, Niels Aarsæther, Amanda Littlewood-Evans, Jeanette Wood, James B. Lorens

**Affiliations:** 1 Department of Biomedicine, University of Bergen, Bergen, Norway; 2 Oncology Research, Novartis Institutes for BioMedical Research, Basel, Switzerland; Karolinska Institutet, Sweden

## Abstract

**Background:**

Blood vessels comprise endothelial cells, mural cells (pericytes/vascular smooth muscle cells) and basement membrane. During angiogenesis, mural cells are recruited to sprouting endothelial cells and define a stabilizing context, comprising cell-cell contacts, secreted growth factors and extracellular matrix components, that drives vessel maturation and resistance to anti-angiogenic therapeutics.

**Methods and Findings:**

To better understand the basis for mural cell regulation of angiogenesis, we conducted high content imaging analysis on a microtiter plate format *in vitro* organotypic blood vessel system comprising primary human endothelial cells co-cultured with primary human mural cells. We show that endothelial cells co-cultured with mural cells undergo an extensive series of phenotypic changes reflective of several facets of blood vessel formation and maturation: Loss of cell proliferation, pathfinding-like cell migration, branching morphogenesis, basement membrane extracellular matrix protein deposition, lumen formation, anastamosis and development of a stabilized capillary-like network. This phenotypic sequence required endothelial-mural cell-cell contact, mural cell-derived VEGF and endothelial VEGFR2 signaling. Inhibiting formation of adherens junctions or basement membrane structures abrogated network formation. Notably, inhibition of mural cell VEGF expression could not be rescued by exogenous VEGF.

**Conclusions:**

These results suggest a unique role for mural cell-associated VEGF in driving vessel formation and maturation.

## Introduction

Mural cells, a peri-endothelial localized mesenchymal cell type including vascular smooth muscle cells and pericytes, exert a dominant effect on endothelial cell behavior, driving vessel maturation, regulating vascular function and influencing responsiveness to anti-angiogeneic therapeutics [1]–[4]. Maturation of blood vessels is a stepwise transition from an actively growing vascular bed to a quiescent functional network [5]. This entails strict temporal and spatial coordination of endothelial cell signaling pathways that govern proliferative, migratory and morphogenic endothelial phenotypes [6]. The recruitment of mural cells to the abluminal surface of nascent blood vessels is a key prerequisite for vessel maturation. Mural cells define a context comprising heterotypic cell-cell contacts, ECM deposition and soluble factors that inhibits endothelial proliferation, maintains capillary diameter, regulates blood flow and provides survival signals [7].

Prior to mural cell coverage, nascent vessels are susceptible to remodeling and VEGF-signaling inhibitors [1]. Genetic and pharmacological inhibition of PDGFR-β reduces mural cell recruitment to growing vessels and is associated with exacerbated angiogenesis, endothelial hypertrophy and irregular, enlarged vessels [8], [9]. Interestingly, endothelial cell VEGF expression can alter mural cell responsiveness to PDGF via growth factor crosstalk, modulating mural cell recruitment and mural function [10]. Abnormal mural cell interactions in tumor vasculature contribute to the presence of non-perfused vessels, aberrant vessel size, loss of the hierarchical vessel organization and sensitivity to anti-angiogenic agents [11].

Heterotypic cell-cell contact at interdigitations between endothelial cells and mural cells provides a unique presentation context for paracrine factors such as VEGF and angiopoietins that regulate endothelial cellular responses [4], [10], [12]. Mural-endothelial cell-cell contact leads to TGF-β1 activation, which in turn inhibits endothelial cell proliferation, while inducing differentiation of mesenchymal stem cells into pericytes [13], [14]. An important system governing vessel maturation is endothelial Tie2 receptor activation by mural cell Ang-1 [15], [16]. Angiopoietin-Tie2 signaling, where endothelial Ang-2 competes with mural cell-derived Ang-1, controls endothelial responsiveness to VEGF and angiogenic remodeling [17], [18]. Indeed, differential requirements for VEGF in immature and mature vessels accounts for the observed vascular “normalizing” effect of VEGF signaling inhibitors in clinical use [19], [20].

In spite of the central role that mural cells play in determining vascular maturation and anti-angiogenic therapeutic responses, our understanding of the molecular mechanisms mediating these effects is incomplete. *In vitro* organotypic angiogenesis models, such as the co-culture of primary endothelial (umbilical vein EC or microvascular EC) and mural cells (pericytes, vascular smooth muscle cells (vSMC), fibroblasts, mesenchymal stem cells) in two- and three-dimensional (imbedded in extracellular matrix) assays [21], have been used to study heterotypic interactions required for EC-mural cell crosstalk [6], [13], [14], [18], [22]–[28]. We adapted EC-mural cell co-culture to a microtiter plate format to facilitate high throughput interrogation of mural cell-dependent regulation of endothelial cell behaviors by combined chemical genetic manipulation and quantitative high content imaging techniques. Using this approach we reveal that this high throughput screening (HTS)-compatible EC-mural cell co-culture system recapitulates a remarkable assortment of angiogenic endothelial cell behaviors. Mural cells define a context in co-culture that enables an endothelial cellular program resulting in formation of a capillary-like network, via a predictable sequence of phenotypic changes reflective of several facets of angiogenesis and vessel maturation: i) loss of EC proliferation; ii) pathfinding cell migration; iii) adherens junction formation; iv) branching morphogenesis and network formation; v) vascular basement membrane-like formation, collagen IV/XVIII ensheathment; vi) patent lumen formation; vi) anastamosis and vii) network stabilization. Our findings emphasize a key role for mural cell presentation of VEGF to drive vessel formation and maturation.

## Results

### ECs co-cultured with vSMCs or mesenchymal stem cells form capillary-like networks

**Figure 1:**
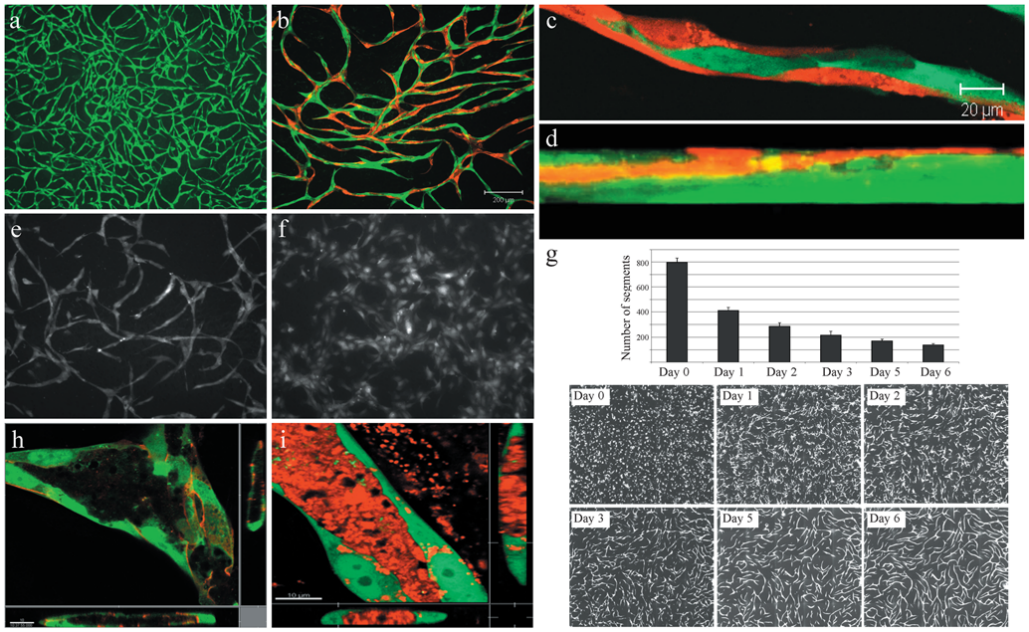
Endothelial cells in co-culture with vascular smooth muscle cells generate capillary-like networks. (A) Live cell fluorescence microscopy of GFP-expressing HUVEC cell capillary-like network (green) after 5 days in co-culture with PA-vSMC (unlabeled). (B) Mixed RFP- and GFP-expressing HUVEC (1∶1) in co-culture with PA-vSMC (unlabeled) at Day 9 demonstrate extensive interaction between ECs within network. (C) Two-color HUVEC networks (9 days) comprise uniform elongated, inter-digitating ECs. (D) Confocal fluorescence microscopy analysis (z-stack) of RFP-expressing HUVEC and GFP-expressing PA-vSMC co-cultures show cells self-organize into distinct layers with ECs residing atop a confluent vSMC layer. (E) Cells grown in co-culture allowing cell-cell contact results in a capillary-like network. (F) Co-cultures in separated by a porous membrane in transwell chambers do not form a network. (G) Temporal image analysis during 6 days of EC network formation shows a decrease in the number of non-networked cells as EC connectivity is established and stabilizes. (H) Lumens form at branch points (inset: z-stacks) and throughout the EC network. (I) TRITC-dextran (10 000 MW) concentrates into patent lumens in Day 9 endothelial networks (inset: z-stacks).

**Figure 2:**
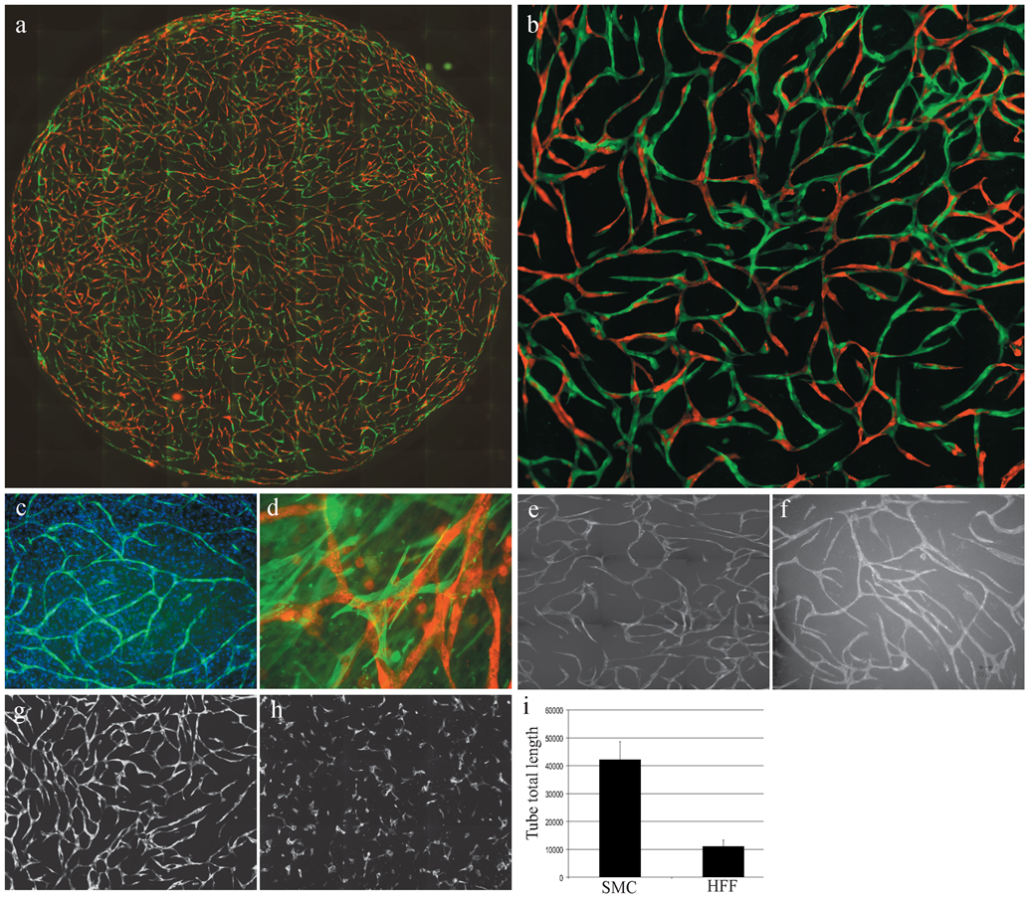
Endothelial- mesenchymal stem cell co-culture generates capillary-like networks. (A) Live cell fluorescence microscopy whole well image 9×11 montage of RFP-expressing HUVEC - GFP-expressing human dermal microvascular endothelial cell (HuMVEC) – PA-vSMC (unlabeled) tri-culture network at Day 6 (10X objective) (B) Live cell fluorescence microscopy image 9×11montage of interdigitating HUVEC (RFP, red) and HuMVEC (GFP, green) Day 6 tri-culture with vSMC (40×objective). (C) Hoechst nuclear staining of a Day 5 co-culture showing the GFP-expressing endothelial network (green) amid a confluent PA-SMC layer (blue). (D) Confocal microscopy analysis of anti-α-SMA-stained PA-SMC (green) extending filopodia that co-localize with multiple RFP-expressing HUVEC (red) in the vessel-like network. (E) Live cell fluorescence microscopy of GFP-expressing HuMVEC capillary-like network after 5 days in co-culture with PA-vSMC. (F) Live cell fluorescence microscopy of GFP-expressing HUVEC capillary-like network after 5 days in co-culture with primary human bone marrow-derived mesenchymal stem cells (MSC). Comparison of GFP-expressing HUVEC co-cultured with PA-SMC (G) and GFP-expressing HUVEC co-cultured with primary human foreskin fibroblast cells (H). Image analysis shows that HFF are unable to support endothelial network formation (I).

To study how mural cells affect endothelial cell behavior we used a genetically and pharmacologically tractable *in vitro* model system comprising co-cultured primary human endothelial and vascular smooth muscle (EC-vSMC) cells [29]. During a one-week period primary human ECs co-seeded with vSMCs in microtiter plates undergo a predictable series of phenotypic changes that result in a stable, interconnected network (Figure 1A-B). Elongated ECs interdigitate to form capillary-like tubular structures of uniform diameter (Figure 1C). Co-cultured EC and vSMC cells rapidly self-organize into two distinct layers, vSMCs forming a confluent layer on the culture dish surface and ECs above (Figure 1D). Impeding this cellular self-organization by pre-attaching ECs to the culture vessel surface followed by vSMC plating, blocked EC network formation (data not shown). Heterotypic cell contact was required as neither vSMC-conditioned medium alone nor co-culture in transwell chambers resulted in EC network formation (Figure 1E-F; data not shown). vSMC monolayers fixed by a variety of methods (e.g cross-linking agents, freeze-thaw) also do not induce EC network formation (data not shown). Time-lapse video fluorescence microscopy analysis revealed that the formation of EC networks progresses through distinct phases (Figure 1G and Video S1). ECs immediately stop dividing and during the first 48 hours become elongated and highly migratory. Migrating ECs form “cell trains” and branched junctions eventually enlisting nearly every cell into the final network (Figure 1G). Once formed, these networks stabilize, ceasing migration (Video S2) and form a patent lumen structure that concentrates exogenous dextran (Figure 1H-I). There is no observable EC apoptosis during this process (Video S1) and stabilized EC networks can been maintained in culture for several weeks (data not shown). Both primary human microvascular endothelial cells (HuMVEC) and primary human umbilical cord vein endothelial cells (HUVEC) formed indistinguishable networks in co-culture with vSMC (Figure 2A-B). Endothelial vessel-like structures assemble amid a confluent monolayer of vSMC (Figure 2C). Confocal microscopy analysis shows that vSMC extend filopodia that co-localize with multiple endothelial cells at various points both above and below the focal plane (Figure 2D). Endothelial networks could also be induced by co-culture with human bone marrow-derived mesenchymal stem cells (Figure 2E-F). In contrast, normal human dermal fibroblasts did not support network formation in co-culture with EC (Figure 2G-I). This indicates that the mural cell-defined context provides a specific set of signals to drive the formation of a stable mature capillary-like network.

### ECs co-cultured with vSMCs quiesce, undergo Rac1-dependent morphogenesis and form adherens junctions during network formation

**Figure 3:**
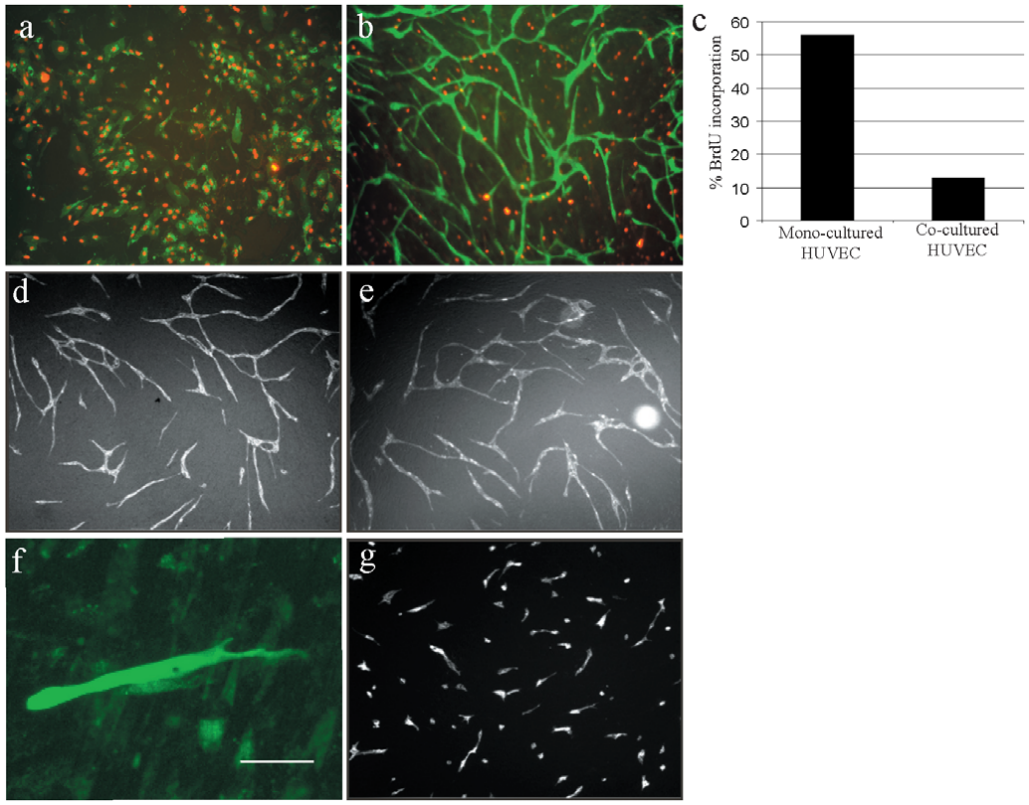
Vascular smooth muscle cells inhibit endothelial cell proliferation and drive Rac1-dependent morphogenesis. (A) GFP-expressing HUVEC grown in mono-culture exhibit extensive BrdU-incorporation (red). (B) In constrast, co-cultured GFP-expressing HUVEC do not incorporate BrdU, while PA-vSMC SMC divides until confluency. (C) Flow-cytometry analysis showed that HUVEC cells in co-culture have a 4.4-fold decreased proliferation activity compared to a HUVEC mono-culture. Data shown is representative of three individual experiments. (D) Over-expression of the cell cycle inhibitor p21 (GFP-p21) in co-cultured HUVEC did not affect EC network formation relative to GFP-expressing HUVEC control (E). (f) A representative GFP-expressing HUVEC in co-culture at 24 hours post-seeding showing a cell migratory morphology (scalebar = 50 µm). (G) Over-expression of dominant negative Rac1 (GFP-Rac1N17) in co-cultured HUVEC potently inhibited cell morphogenesis and EC network formation.

Time-lapse fluorescence microscopy analysis of GFP-expressing EC in co-culture revealed no apparent cell division (Video S1). Analysis of BrdU-incorporation in EC-vSMC co-culture demonstrated that while vSMCs divide in co-culture until confluent, ECs show low BrdU-levels (Figure 3A-C). Indeed, over-expression of the cell cycle inhibitor p21 in ECs did not affect network formation (Figure 3D-E). Co-cultured ECs display a polarized, migratory phenotype during network formation (Figure 3F). Expression of dominant negative Rac1 (Rac1N17) strongly inhibited morphogenesis and network formation emphasizing the importance of Rac1-dependent cytoskeletal rearrangements (Figure 3G).

**Figure 4:**
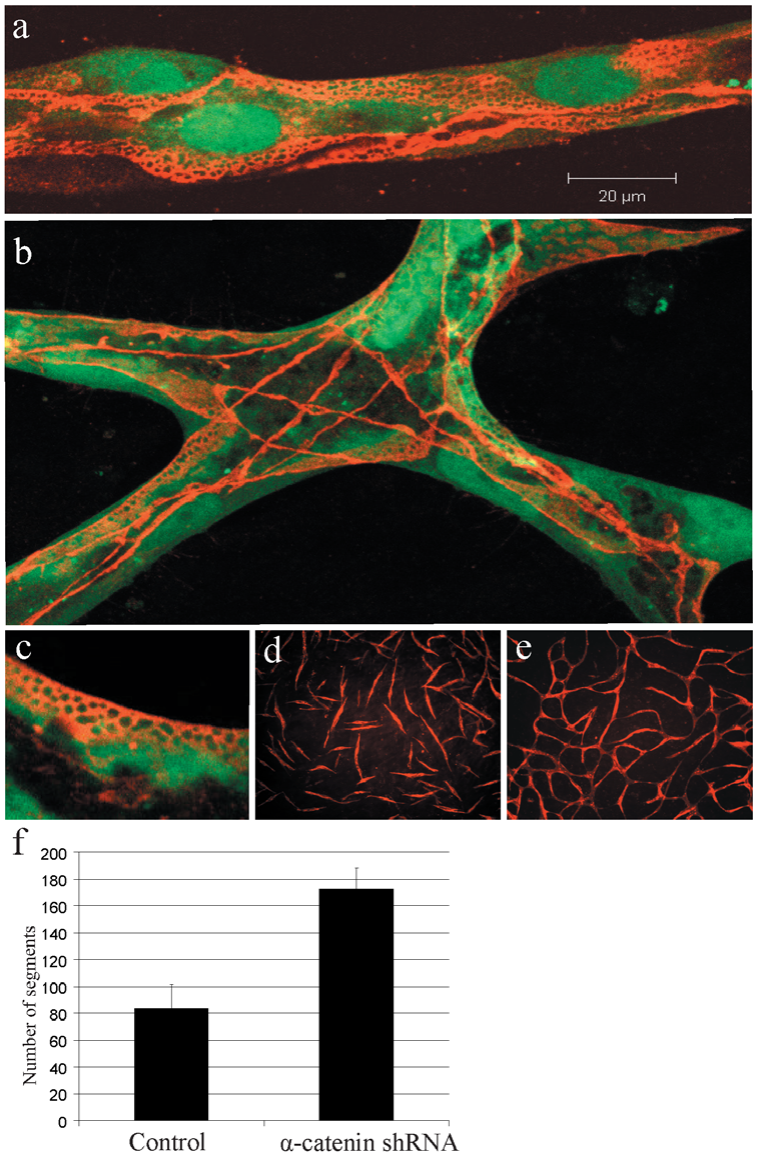
Endothelial cell networks form adherens junctions. (A) Adherens junctions visualized by VE-cadherin immunofluorescence (red) delineate the border of interdigititated HUVEC cells (green) in 6 day capillary-like networks. (B) Confocal imaging (collapsed z-stack) of a network branch point where VE-cadherin immunofluorescence defines borders of layered, intersecting endothelial cells. (C) VE-cadherin immunofluorescence showed a lace-like structure. (D) Retroviral vector shRNA-mediated knockdown of α-catenin in co-cultured HUVEC results in inhibition of network formation. Data shown is representative of three independent shRNAs targeting α-catenin. (E) Control shRNA expressing co-cultured HUVECs generate a capillary-like network. (F) Image analysis of fluorescence microscopy images showed a 2-fold increase in distinct HUVEC (segments) detected at 72 hours post co-culture seeding.

Adherens junctions represent the major class of homotypic endothelial cell-cell interactions [30]. VE-cadherin is a specific marker for vascular endothelial cells adherens junctions, and immunofluorescence analysis demonstrated extensive VE-cadherin-catenin complex formation in co-culture induced EC networks (Figure 4A-B). Notably, VE-cadherin complex structures displayed a distinct “lacelike” appearance in co-cultured EC, perhaps due to increased interdigitation and junctional tightening (Figure 4C) [31]. The functional significance of adherence junction formation in network formation was ascertained by RNAi knockdown of α-catenin (Figure 4D-F; Figure S1). EC expressing an shRNA targeting α-catenin failed to generate an interconnected capillary-like network in co-culture, instead forming single, elongated, unconnected ECs that were stable (Figure 4D). α-catenin knockdown in HUVEC did not alter quiescence or enhance cell death in co-culture (data not shown). Thus homotypic EC connectivity via adherens junction formation is separable from vSMC-induced endothelial quiescence and morphogenesis.

Together these results indicate that while formation of a single interconnected network relies on the formation of EC-EC adherens junctions, these junctions are not required for the endothelial cells to undergo extensive and stable changes in cell morphology.

### ECs co-cultured with vSMCs form vascular basement membrane-like structures

**Figure 5:**
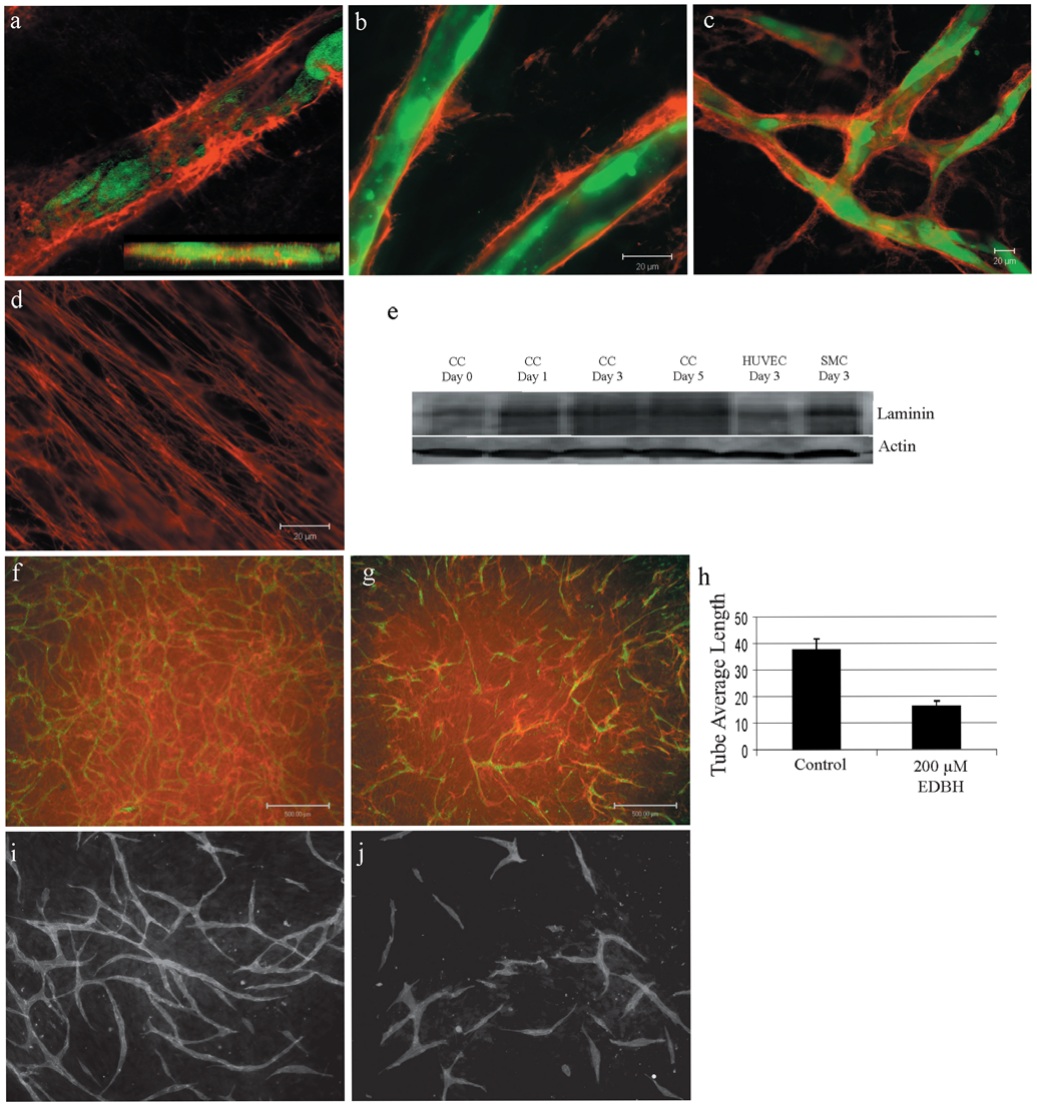
Endothelial cell networks are stabilized by basement membrane protein deposition. (A) Collagen type IV (red) is deposited at GFP-expressing HUVEC cells in co-cultures, completely enveloping the endothelial cells (confocal Z-stack, inset). (B) Collagen type IV and (C) collagen type XVIII have similar EC-associated deposition patterns. (D) SMC-associated laminin expression, 100×objective. (E) Western blot analysis shows that laminin deposition increases during co-culture. (F) Immunofluorescence analysis of collagen IV (red) expression in 72 hour vehicle-treated co-cultures with GFP-expressing HUVEC cells and PA-vSMC (unlabelled). (G) Inhibition of collagen type IV synthesis/maturation by EDBH treatment reduces collagen IV deposition and tube formation. (H) Quantification shows that EDBH-treatment reduces EC network tube average length. (J) Co-cultures incubated in ascorbic acid-deficient medium inhibited EC network formation compared to control (I).

Formation of the vascular basement membrane is a hallmark of vessel maturation [3]. We therefore interrogated EC-vSMC co-cultures for the presence of basement membrane matrix protein deposition. Confocal immunofluorescence analysis demonstrated the presence of extensive collagen IV protein structures that completely ensheathed EC networks at 72 hours (Figure 5A-B). Collagen IV was not expressed by SMCs or ECs in monoculture (data not shown), suggesting an induced synthesis and local collagen IV deposition in co-culture. Collagen XVIII, a specific constituent of vascular basement membranes showed a similar peri-endothelial enriched localization pattern in co-culture (Figure 5C). Other ECM proteins, laminin (Figure 5D) and fibronectin (data not shown) localized at the vSMC layer. Laminin expression increased over time in the EC-vSMC co-culture (Figure 5E).

To determine whether the formation of a collagen-rich vascular basement membrane-like structure was necessary to stabilize EC capillary-like networks, co-cultures were treated with a proline hydroxylase inhibitor (EDBH) or starved for ascorbic acid to inhibit collagen assembly and synthesis respectively (Figure 5F-J). These treatments strongly reduced EC-associated collagen deposition, disrupting EC connectivity, reducing tube length and abrogating EC networks (Figure 5J). Hence formation of a vSMC-induced vascular basement-like structure contributes to the overall EC network stability.

### ECs require VEGF-R2 and vSMC-associated VEGF to form a capillary-like network

**Figure 6:**
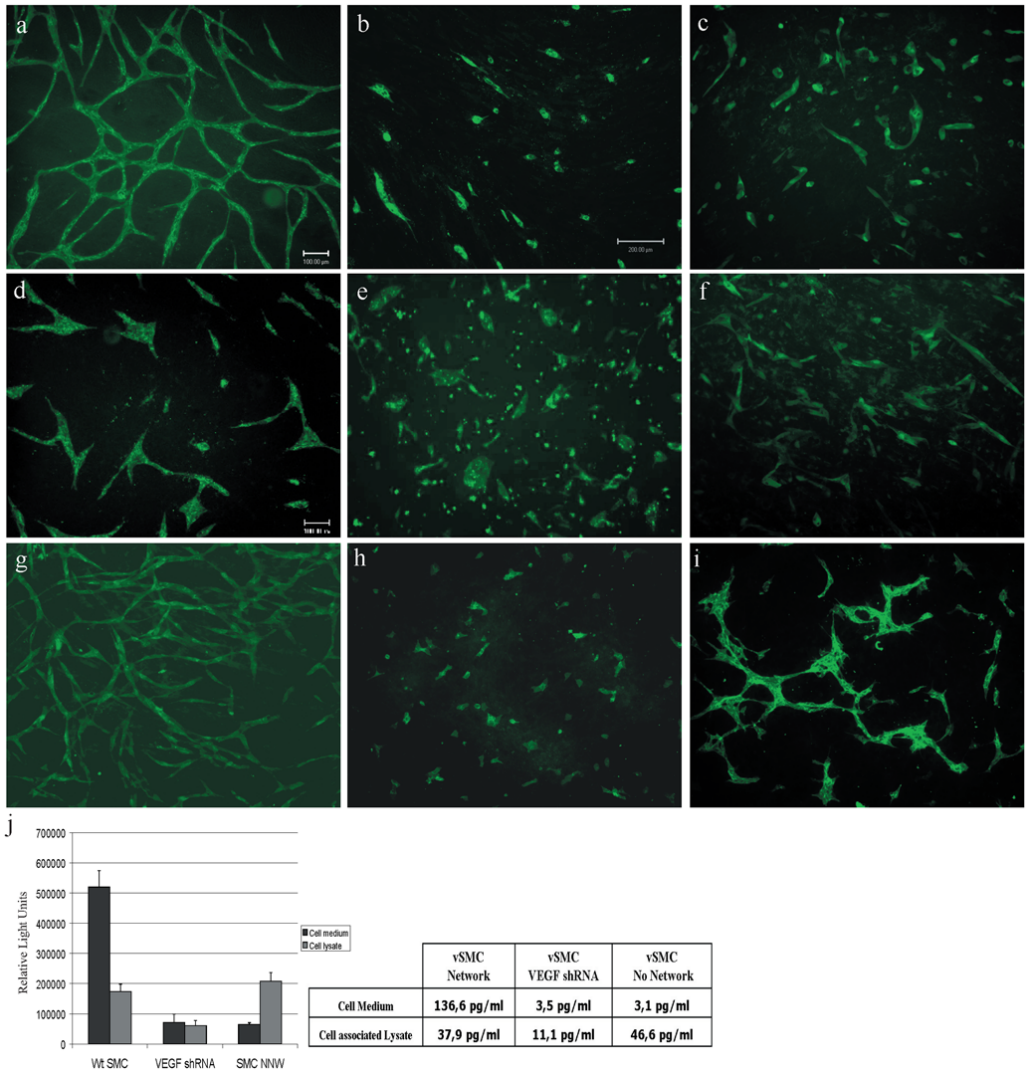
Vascular smooth muscle cell-derived VEGF is required for endothelial capillary-like network formation. (A) Fluorescence microscopy image of GFP-expressing HUVEC and PA-vSMC co-cultures at day 3 post-seeding. Treatment of EC-vSMC co-cultures with VEGF blocking antibody (Avastin, B) or small molecule VEGFR inhibitor (PTK787/ZK, C) block EC network formation. (D) Transduction of HUVEC with a shRNA targeting VEGFR-2 retroviral vector also strongly inhibited network formation. (E) A human PA-vSMC isolate (NNW) that has very low VEGF production and secretion does not support EC network formation. (F) shRNA-mediated knockdown of VEGF levels in human PA-vSMC inhibits network formation. (G) Overexpression of VEGF165 in PA-vSMC did not affect EC network formation. (H) SV40-immortilized mouse embryonic fibroblasts (SV40-MEF) were unable to drive EC network. (I) HUVEC cells co-cultured with SV40-MEF cells that overexpress VEGF165 form incomplete networks. (J) ELISA quantification of VEGF levels from different vSMC cells.

Previous studies demonstrated the requirement for VEGF to drive endothelial network formation in different systems including EC-vSMC co-cultures [22], [32]. Indeed, network formation in EC-vSMC co-cultures is strongly inhibited by treatment during the initial 48 hours with different VEGF-signaling inhibitors (Figure 6A-C). Further, shRNA-mediated knockdown of VEGF-R2 in ECs blocked network formation (Figure 6D; Figure S1). Timelapse video fluorescence microscopy of PTK787/ZK-treated co-cultures demonstrated that ECs continue to migrate but do not elongate nor form EC-EC junctions (Video S3). This is congruent with the notion that ECM proteins alone are sufficient to induce cell migration but vSMC-VEGF induced signaling via endothelial VEGF-R2 is required for network formation [23].

As co-cultures conducted in growth factor-rich or poor (i.e. 2% serum only) conditions both displayed network formation (data not shown), we reasoned that VEGF was provided by vSMCs. Indeed, RT-PCR (Figure S2) and ELISA (Figure 6J) on cultured vSMCs demonstrated that VEGF165 is a predominant isoform, expressed at 135 pg/ml per 700 000 cells/day. shRNA-mediated knockdown of vSMC VEGF-expression (3.5 pg/ml per 700 000 cells/day) completely blocked EC capillary-like network formation (Figure 6F). Similarly, vSMC isolates that do not secrete VEGF165 (<3 pg/ml per 700 000 cells/day) also do not support EC network formation (Figure 6E). Importantly, the inhibitory effects of vSMC-VEGF knockdown on network formation phenotype could not be rescued by co-culture in optimal endothelial growth medium that contains VEGF, bFGF and EGF (EGM-2 medium) nor by addition of conditioned native vSMC medium (Figure 6E-F; data not shown), conditions that readily support migration and proliferation of EC cultures. Conversely, we over-expressed VEGF_165_ in vSMC (3238 pg/ml per 700 000 cells/day) by retroviral transduction and evaluated the effects on EC in co-culture. As shown in Figure 6G these EC-vSMC^VEGF-High^ co-cultures displayed normal network formation. Interestingly, retroviral vector expression of VEGF165 in fibroblasts was insufficient to drive the EC capillary-like network formation observed for vSMC-EC co-cultures, instead inducing EC morphology changes characterized by extended filopodia (Figure 6H-I). These results demonstrate that vSMC-associated VEGF is required to drive EC capillary-like network formation and that enhancing this expression does not lead to the formation of an excessive or less mature EC network. This indicates that mural cells can define a context that limits the extent of VEGF-induced angiogenesis and promotes the formation normal vessels.

## Discussion

Mural cells exert a dominant effect on endothelial cell behavior that is necessary for vessel maturation and function, and that determines responses to anti-angiogenic therapeutics [2], [3]. We report here that primary human mesenchymal cell types, vascular smooth muscle and mesenchymal stem cells, induce functional and morphological changes in primary endothelial cells (HUVEC, HuMVEC) that lead to a uniform, stabilized capillary-like structure, enveloped by vascular basement membrane matrix proteins and maintaining a patent lumen, hence recapitulating major structural components of blood vessels in a high throughput-high content imaging compatible format.

Mural cell interactions attenuate endothelial proliferation, a prerequisite for maturation and stability. Reduced mural cell recruitment to sprouting endothelial cells is associated with vessel hypertrophy *in vivo* [8]. Studies employing *in vitro* co-culture systems demonstrated that mesenchymal cells induce endothelial quiescence by direct cell-cell contact, mediated in part by activation of TGFβ [18], [24]. Congruent with these reports we demonstrate that EC proliferation is inhibited in co-culture with vSMC and that enforced cell cycle arrest by p21 cyclin-dependent kinase inhibitor expression has no effect on endothelial network formation. Instead endothelial cells become highly migratory and morphogenic in the presence of mesenchymal cells. Time-lapse fluorescence confocal microscopy analysis shows that endothelial cells engage in extensive pathfinding-activity during the first 72 hours, forming motile cell trains that integrate into fully interconnected anastamosed endothelial networks.

Several studies using various *in vitro* angiogenesis systems (e.g. co-culture, 3-D collagen, Matrigel) have shown that VEGF is required for endothelial cell network formation [22], [32]. Consistent with this, treatment with a VEGFR tyrosine kinase inhibitor (PTK787/ZK), RNAi knockdown of endothelial VEGF-R2 or vSMC-VEGF expression completely abrogates co-culture-induced endothelial network formation. Time-lapse fluorescence videomicroscopy analysis of PTK787/ZK-treated co-cultures demonstrated that EC remain viable and actively migrate, likely due to vSMC matrix protein expression (fibronectin, collagen I), but do not elongate or form stable cell-cell contacts. VEGF activates Rho-family small GTPases that induce endothelial cytoskeletal rearrangements that are required for cell elongation and vessel formation [25], [33]. Indeed, we show that expression of dominant negative Rac1 potently inhibits EC morphology changes in co-culture with vSMC. In contrast, targeted disruption of adherens junction formation via shRNA knockdown of endothelial α-catenin blocked the formation of an interconnected endothelial cell network, however without influencing EC elongation.

Assembly of the laminin and collagen IV-rich vascular basement membrane is a critical event in stabilizing newly formed blood vessel [23]. This peri-endothelial matrix requires proteins from both EC and mural cells to form and is hence a key result of mural-EC interactions. We show that important aspects of the basement membrane assembly are uniquely recapitulated in vSMC-EC co-culture: In particular, the induction and local deposition of collagen IV and vascular-specific collagen XVIII that completely ensheaths endothelial cells is not previously reported. Reciprocally there is a marked increase in vSMC laminin expression, which may also contribute to the quiescence of EC [23]. Basement membrane assembly entails collagen IV incorporation into laminin polymers via nidogens [34]. Importantly we demonstrate that this matrix protein structure is functionally significant as inhibitors of collagen synthesis block network formation. Hence the vSMC-EC co-culture system represents a novel *in vitro* model to study vascular basement membrane assembly.

We demonstrate a singular requirement for mural cell-derived VEGF in generating a stable capillary-like network formation. RNAi knockdown of smooth muscle cell VEGF expression phenocopied the effect of VEGF signaling inhibitors. Surprisingly, neither the presence of VEGF, bFGF, EGF (EGM-2 medium) nor conditioned vSMC medium rescued the loss of SMC VEGF expression. This indicates that VEGF must be associated with the smooth muscle cells in a specific context in order to trigger EC morphogenesis. VEGF165, the major isoform expressed by vSMC, is retained at the cell surface by binding heparin sulfate proteoglycans (HSPG) [26]. HSPG-bound VEGF165 activates VEGF-R2 and the co-receptor Nrp1, generating a unique VEGF-R2 signaling cascade [35], a plausible mechanism for this vSMC-association dependent signaling. We show that overexpression of VEGF165 in vSMCs did not affect EC network formation suggesting that the mural-VEGF presentation context is saturable and unaffected by pathological VEGF levels that can lead to abnormal blood vessels and hemangiomas [36].

Mural cells have been shown to express VEGF *in vivo* [26], [37]. VEGF and other growth factors are found at interdigitations between endothelial cells and mural cells, a unique context for mural cell-associated VEGF presentation [12]. Growth factor receptor crosstalk is an important mechanism regulating EC-pericyte interactions [10]. D'Amore and colleagues suggested that pericyte-expressed VEGF may serve to transiently stabilize nascent vessels during basement membrane assembly and lumen formation via juxtacrine VEGF signaling and heterotypic cell-cell contact that replaces tissue-derived VEGF (e.g. from astrocytes in the developing retina) [26]. This switch from tissue-derived to mural cell-derived VEGF could promote vessel maturation and ultimately lead to VEGF-independence. Indeed, mural cell maturation factors, in particular Ang1, have been shown to abolish endothelial VEGF responsiveness to tissue-derived VEGF [16], [18]. Modulating Ang-Tie2 signaling (by either gain- and loss of function) did not affect network formation in EC-vSMC co-culture (data not shown). Together with our observation that soluble VEGF cannot substitute for mural cell-expressed VEGF this supports the notion that these VEGF presentation forms are functionally distinct [35]. Our recent results indicate that the vSMC co-culture-context leads to altered endothelial cell VEGF-receptor signaling and that this is necessary to achieve a stable capillary-like network (Evensen et al., in preparation).

In conclusion, our findings emphasize a unique role for mural cell-derived VEGF in driving blood vessel maturation and define an informative high throughput-high content imaging-compatible experimental system for studying blood vessel formation.

## Materials and Methods

### Cell culture

Human umbilical vein endothelial cells (HUVEC), pulmonary artery smooth muscle cells (Pa-vSMC), human adult dermal microvascular endothelial cells (HUMVEC) and human mesenchymal stem cells (hMSC) were purchased from Lonza (C2517A, CC2581, CC2543, PT2501). To simplify imaging, early passage HUVEC cells were infected with retrovirus carrying a GFP or RFP-expressing construct. Cells were maintained in culture in the supplier's recommended complete medium (EGM-2, SmGM-2, EGM-2MV and MSCGM respectively) at 37°C, 5% CO_2_. The growth medium was changed every third day and cells were passaged prior to reaching confluence. The maximum passage number used for experiments was 8 (HUVEC), 12 (PaSMC), 8 (HUMVEC), 6 (hMSC). Primary human foreskin fibroblasts were provided by Dr. Ola Hammarsten, Sahlgrenska University Hospital, Gothenburg, Sweden. SV-40 immortalized mouse embryonic fibroblasts were provided by Prof. Donald Gullberg, University of Bergen, Norway.

### EC-mural cell co-culture assay

PaSMC and HUVEC were seeded together, centrifuged briefly at 200 g to achieve an even distribution of cells and cultured in EGM-2 for at least 72 hrs to allow network formation. For longer periods, culture medium was changed every third day. Cell numbers and culture volume were as follows (per well): 96-well plates: 5×10^4^ PaSMC, 10×10^3^ HUVEC, 200 µl EGM-2; 6-well plates: 8×10^5^ PaSMC, 1.5×10^5^ HUVEC, 2 ml EGM-2; transwells: 1.75×10^5^ PaSMC, 6×10^3^ HUVEC, 1 ml EGM-2; 8-well chamber slides: 1.4×10^5^ PaSMC, 3×10^4^ HUVEC, 250 µl EGM-2. HUMVEC and hMSC experiments were conducted similarly using EGM-2MV as the culture medium.

### Microscopy and High content imaging

Confocal images were acquired on a Zeiss LSM 510 Meta. A Zeiss Axiovert S100 fluorescence microscope with a Roper Quantix digital camera was used for conventional fluorescence and brightfield microscopy. For quantitative analysis of the co-cultures a BD Pathway 855 bioimaging system (BD Biosciences, San Jose, Ca) was used for automated high throughput imaging. Statistical analysis of acquired images was done with BD Image Data Explorer software. Images were acquired as 3×3 montages using a 10×lens. Background subtraction, noise reduction (rolling ball) and image thresholding were performed using the AttoVision v1.6.1 software supplied by BD Biosciences. Statistics on tube branch lengths, and number of branch points per region of interest were obtained using the “Tube Formation” image analysis module of AttoVision v1.6.1. *Tube total length* is the total number of pixels comprising the network in the image field. *Tube average length* is the total number of pixels comprising the network in the image field divided by number of unconnected tube segments. *Number of segments* is the number of unconnected segments in the image field.

### Immunohistochemistry

#### BrdU staining

BrdU (10 µM) was added 4 hrs after seeding cells and incubated overnight. Cultures were trypsinated, fixed (1.6% PFA, 25 mins, room temperature) and permeabilized (100% methanol, 2 hrs, 4°C). BrdU was visualized by staining with monoclonal anti-BrdU (Sigma, B8434, 1∶500 dilution in PBS/2% FBS) followed by an allophycocyanin conjugated goat anti-mouse IgG secondary (Molecular Probes, A865, 1∶3000 dilution). BrdU staining was quantified by flow cytometry (10 000 events) on a FACSCalibur (BD Biosciences) and performed as three independent experiments.

#### VE-cadherin staining

Cells in 8-well chamber slides were washed (PBS), fixed (4% PFA, 25 minutes, room temperature) and permeabilized and blocked (PBS/0.3% Triton X100/5% normal goat serum, 1 hr, room temperature). VE-cadherin was visualized with rabbit anti-VE-cadherin antibody (Santa Cruz Biotechnology, SC9989, 1∶200 in PBS/0.3% Triton X100/2% FBS, overnight, 4°C). Cells were washed (PBS, 3×5 mins), treated with secondary antibody (Molecular Probes, A21244, Alexa647-conjugated goat-anti-rabbit IgG, 1∶3000, 2 hrs, room temperature in the dark) and washed again (PBS, 3×5 mins).

#### Extracellular matrix staining

As for VE-cadherin staining using primary antibodies diluted 1∶200 in PBS/2% FBS and secondary antibodies diluted 1∶3000 in PBS/2% FBS (Molecular Probes, A21123, Alexa546-conjugated goat anti-mouse IgG). Primary antibodies were: Monoclonal mouse anti-Collagen type IV antibody (Chemicon; MAB3326), rabbit anti-Laminin (Chemicon; AB19012), and monoclonal mouse anti-collagen type XVIII (a kind gift from Ritva Heljasvaara and Taina Pihlajaniemi, University of Oulu, Finland).

#### Lectin staining

Cells were fixed with 4% PFA as above. HUVEC in co-culture were selectively stained with FITC/TRITC-UAE 1 lectin (Sigma; L9006/L4889, 1∶1000 dilution in PBS, 45 minutes, room temperate in the dark) and washed (PBS, 3×5 mins).

#### Dextran staining

Mature day 8 co-cultures were incubated with Texas Red-conjugated 10,000 MW dextran (Molecular Probes, D1828, 0.5 mg/ml, overnight).

### RNA interference

RNA interference was achieved using retrovirally expressed shRNAs. Complementary shRNA oligos (PAGE purified, TAGC Copenhagen) were annealed and ligated into the MMLV-derived retroviral shRNA expression vector L071 RRI-Green or L087 RRI-Red (Entrez:EU424172, EU424173). These vectors also express a puromycin resistance marker and GFP or RFP respectively. Transduced cells were isolated by FACS utilizing fluorescent protein reporter expression. Final hairpin sequences were:

### α -catenin shRNA

GCAGATGTCTACAAATTACTTGTTCAGCTctggtcAGCTGAACAAGTAATTTGTAGACATCTGC

### VEGFR-2 shRNA oligo

GAACATTTGGGAAATCTCTTGCAAGCTAgaagcttgTAGCTTGCAAGAGATTTCCCAAATGTTC

### VEGF shRNA

GTGGTGAAGTTCATGGATGTCTATCAGCGctggtcCGCTGATAGACATCCATGAACTTCACCAC

### GFP, RFP, VEGF, RacN17 and p21 overexpression

Overexpression of proteins in cells was performed by transfection of a 293T packaging cell line with a retroviral expression vector containing the DNA of interest. Virus was harvested 24–48 hrs after transfection in medium suited for the target cells. Infection was performed by filtering the virus containing medium, addition of proteamine sulphate, and transferring of the virus containing medium to the target cells which were incubated further for 24 hrs. Infection was ended by changing the medium. Protocol will be given out by request. GFP was expressed from pCGFP [38]. RFP was expressed from pCtdTomato, a derivative of pCGFP with the GFP replaced by tdTomato [39]. RacN17 and p21 were expressed from IRES-GFP retroviral vector [38]. VEGF was co-expressed with GFP and a puromycin resistance marker using a 2A-mediated expression system[40].

### Quantification of VEGF

VEGF in cells and culture medium was quantified using an ELISA kit (R&D systems, Human VEGF QuantiGlo Elisa, QVE00B) and a HIDEX Plate Chameleon luminometer. Samples were prepared as follows: Culture medium from 24 hrs was collected, cell debris removed by centrifugation and samples were stored at −80°C after addition of protease inhibitors (Roche, Complete Protease Inhibitor Cocktail tablets, 11697498). Cells were treated with lysis buffer containing 20 mM Tris (pH 8.0), 150 mM NaCl, 1 mM dithiothreitol, 1% deoxycholic acid, 0.5% sodium dodecylsulfate, 1% Nonidet P-40, and protease inhibitors. Prior to freezing of samples small aliquots were taken out to determine protein concentration.

### Drug treatment of co-cultures

300 µM ethyl-3–4-dihydroxybenzoate (EDBH, Sigma, E24859-5G) was added to the cell suspension before plating and treatment was continued for 72 hrs. Avastin [41] was added identically to a final concentration of 1 µg/ml. PTK787/ZK222584 [42] (Oncology Research, Novartis Institutes for BioMedical Research), a potent inhibitor of vascular endothelial growth factor (VEGF) receptor tyrosine kinases and class III kinases (platelet-derived growth factor (PDGF) receptor beta tyrosine kinase, c-Kit, and c-Fms), was dissolved in dimethyl sulfoxide (DMSO) at a stock concentration of 10 mM and added to co-cultures at a concentration of 100 nM.

## Supporting Information

Figure S1 Retroviral shRNA knockdown in primary endothelial cells. (a) Cell lysates from HUVEC mono-cultures transduced with luciferase shRNA or α-catenin shRNA were analysed by 10% SDS-PAGE and show knock-down of the 102 kDa protein α-catenin. Actin, 42 kDa, was used as a loading control. Results are representative of 3 different shRNA sequences that silence human α-catenin expression. Each of these different α-catenin shRNA sequences engendered the non-branching phenotype shown in Figure 4d. (b) HUVEC transduced with luciferase shRNA or VEGFR-2 shRNA grown in mono-culture were recovered and surface stained for VEGFR-2 levels. HUVEC transduced with VEGFR-2 shRNA (red) show greatly decreased surface levels of VEGFR-2 compared with luciferase shRNA transduced HUVEC (green).
(0.09 MB PDF)

Figure S2 Dominant VEGF isoforms in PA-vSMC. RT-PCR analysis of mRNA isolated from monocultured PA-vSMC. Primers used for nested PCR, VEGF outer: 5′-GGGCAGAATCATCACGA-3′ (156–172) and 5′-CCGCCTCGGCTTGTCACA-3′ (629–612) VEGF inner: 5-′ATCGAGACCCTGGTGGACA-3′ (219–238) and 5′-CCGCCTCGGCTTGTCACA-3′ (629–612). The brackets indicate the positions of primers (Entrez: M32977). Expected sizes of cDNA fragments for the various vascular growth factor (VEGF) transcript alternative splice variants when amplified with outer/or the inner primer pairs: VEGF206 (597 bp/534bp); VEGF189 (546 bp/483 bp); VEGF165 (474bp/411bp); VEGF145 (414 bp/351bp); and VEGF121 (342 bp/279 bp).
(0.09 MB PDF)

Video S1 Time-lapse microscopy analysis of endothelial-mural cell co-culture. Co-cultures (GFP-expressing HUVEC and PA-vSMC) were seeded in 96-well plates and images were acquired every 15th minute on a Zeiss LSM 510 Meta (37°C, 5% CO2). Several time-lapse movies from different timepoints were merged and together span a time range from day 0 to day 9.
(4.33 MB MOV)

Video S2 Time-lapse microscopy analysis of a mature endothelial-mural cell co-culture Co-cultures (GFP-expressing HUVEC and PA-vSMC) were seeded in 96-well plates and images were acquired every 15th minute on a Zeiss LSM 510 Meta (37°C, 5% CO2). Time-lapse is recorded from 216–240 hrs post-seeding of culture and shows a mature quiescent network.
(0.33 MB MOV)

Video S3 Time-lapse microscopy analysis of PTK787/ZK-treated endothelial-mural cell co-culture Co-cultures (GFP expressing HUVEC) were seeded in a 96-well plate and treated with 100 nM PTK787/ZK. A time-lapse was recorded overnight (37°C, 5% CO2) by imaging the cells every 15th minute using a Zeiss LSM 510 Meta.
(0.29 MB MOV)
