# Supplementary figures and images for: Mural Cell Associated VEGF Is Required for Organotypic Vessel Formation
Source: PLoS One. 2009 Jun 4;4(6):e5798. doi: 10.1371/journal.pone.0005798 (PMC2688382; doi:10.1371/journal.pone.0005798)

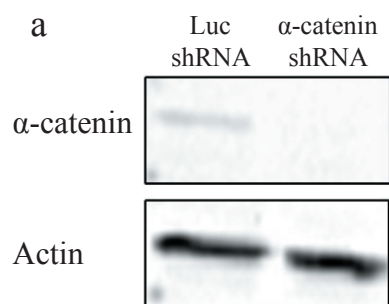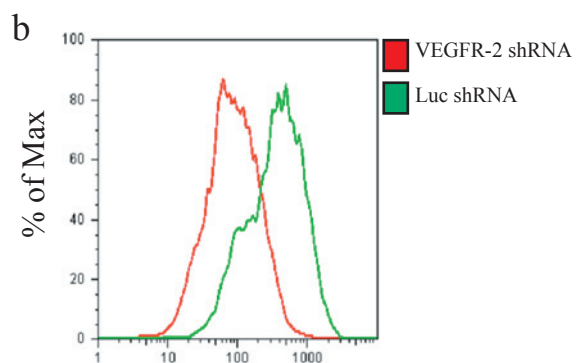

Supplement: Figure S1 — Retroviral shRNA knockdown in primary endothelial cells. (a) Cell lysates from HUVEC mono-cultures transduced with luciferase shRNA or α-catenin shRNA were analysed by 10% SDS-PAGE and show knock-down of the 102 kDa protein α-catenin. Actin, 42 kDa, was used as a loading control. Results are representative of 3 different shRNA sequences that silence human α-catenin expression. Each of these different α-catenin shRNA sequences engendered the non-branching phenotype shown in Figure 4d. (b) HUVEC transduced with luciferase shRNA or VEGFR-2 shRNA grown in mono-culture were recovered and surface stained for VEGFR-2 levels. HUVEC transduced with VEGFR-2 shRNA (red) show greatly decreased surface levels of VEGFR-2 compared with luciferase shRNA transduced HUVEC (green). (0.09 MB PDF) [file pone.0005798.s001.pdf]
