# Supplementary material for: Mural Cell Associated VEGF Is Required for Organotypic Vessel Formation
Source: PLoS One. 2009 Jun 4;4(6):e5798. doi: 10.1371/journal.pone.0005798 (PMC2688382; doi:10.1371/journal.pone.0005798)
Supplement: Figure S2 — Dominant VEGF isoforms in PA-vSMC. RT-PCR analysis of mRNA isolated from monocultured PA-vSMC. Primers used for nested PCR, VEGF outer: 5′-GGGCAGAATCATCACGA-3′ (156–172) and 5′-CCGCCTCGGCTTGTCACA-3′ (629–612) VEGF inner: 5-′ATCGAGACCCTGGTGGACA-3′ (219–238) and 5′-CCGCCTCGGCTTGTCACA-3′ (629–612). The brackets indicate the positions of primers (Entrez: M32977). Expected sizes of cDNA fragments for the various vascular growth factor (VEGF) transcript alternative splice variants when amplified with outer/or the inner primer pairs: VEGF206 (597 bp/534bp); VEGF189 (546 bp/483 bp); VEGF165 (474bp/411bp); VEGF145 (414 bp/351bp); and VEGF121 (342 bp/279 bp). (0.09 MB PDF) [file pone.0005798.s002.pdf]

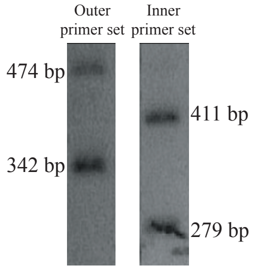

Expected size (bp) of cDNA fragments when amplified with VEGF splice variants primers

	Outer primer pair	Inner primer pair
VEGF 206	597	534
VEGF 189	546	483
VEGF 165	474	411
VEGF 145	414	351
VEGF 121	342	279
